# Efficiency evaluation of urban employee’s basic endowment insurance expenditure in China based on a three-stage DEA model

**DOI:** 10.1371/journal.pone.0279226

**Published:** 2023-03-03

**Authors:** Liping Li, Dongmei Li

**Affiliations:** School of Management, Shanghai University, Shanghai, China; University of Almeria, SPAIN

## Abstract

With the deepening of population aging, the expenditure of basic endowment insurance in China is increasing. The urban employees’ basic endowment insurance(UEBEI) system for is an important part of China’s basic social endowment insurance system, which is the most important institutional guarantee for the basic needs of employees after retirement. It not only relates to the living standards of retired employees but also relates to the stability of the whole society. Especially considering the acceleration of urbanization process, the financial sustainability of the basic endowment insurance for employees is of great significance for safeguarding the pension rights of retired employees and realizing the normal operation of the whole system, and the operation efficiency of urban employees’ basic endowment insurance(UEBEI) fund inevitably becomes the focus of increasing attention. Based on the panel data of 31 provinces in China from 2016 to 2020, this paper established a three-stage DEA-SFA model, and compared the differences of comprehensive technical efficiency, pure technical efficiency and scale efficiency with radar chart, aiming to explore the operating efficiency of the UEBEI in China and how environmental factors affect it. The empirical results show that at present, the overall level of the expenditure efficiency of the UEBEI fund for urban workers is not high, and all provinces have not reached the efficiency frontier level, and there is still a certain space for efficiency improvement. Fiscal autonomy and elderly dependency ratio are negatively correlated with fund expenditure efficiency, while urbanization level and marketization level are positively correlated with fund expenditure efficiency. The regional difference of fund operation efficiency is significant, from high to low, it is East China, Central China and West China. Reasonable control of environmental variables and narrowing of regional economic development and fund expenditure efficiency differences can provide some enlightenment for better realization of common prosperity.

## 1. Introduction

Urban employee’s basic endowment insurance(UEBEI) is a mandatory social insurance, so as to provide urban old people basic and stable life security. The old people can claim after retirement as long as they meet the amount of endowment insurance payment and the relevant number of years of the prescribed conditions. Both employers and employees are required to pay a certain proportion of basic endowment insurance(BEI). The contribution rate of employers in all regions of China will be uniformly lowered to 16 percent [1], with 8 percent paid by individual employees and which transferred to individual accounts. With the continuous improvement of the social security system, China’s BEI system is also developing, and its social security function is constantly enhanced. Especially in the context of the accelerating aging of China’s population [2], it has an important impact on China’s poverty alleviation campaign and the realization of common prosperity [3]. The “14th Five-Year Plan for National Economic and Social Development of the People’s Republic of China and the Outline of the Vision For 2035” (The 14th Five-year Plan) states to improve the mechanism for rationally adjusting of UEBEI, gradually raise the standards of BEI for both urban and rural residents, raise the participation rate of BEI to 95 percent, and develop a multi-tiered and multi-pillar endowment insurance system [4]. Since its establishment, China’s endowment insurance system has always had a strong ability to expend, with a large surplus every year. By the end of 2020, the income of the UEBEI fund nationwide had reached 4.44 trillion RMB and the expenditure had reached 5.13 trillion RMB, with an accumulated balance of 4.83 trillion RMB and 456 million insured people [5].

The UEBEI in China works through funds to maintain and increase its value. With the continuous development of fund services and management level, the payment capacity and balance of UEBEI have been increasing, which has played an important role in narrowing the gap between the rich and the poor, improving people’s livelihood and increasing residents’ happiness [3]. However, there are great differences in the regional balance and payment capacity of UEBEI in different regions [6]. The practice of developed countries shows that the pension system is an effective institutional guarantee to solve the basic living risks of the old people, but it may also become an important source of social risks [7]. With the continuous expansion of fund income and expenditure scale and the number of people covered, the evaluation and analysis of the operating efficiency and influencing factors of insurance expenditure will help to clearly find the deviations between the policy design and actual operation of the UEBEI, and further promote the perfection of the pension system.

## 2. Literature review

### 2.1 UEBEI and the common prosperity

In essence, common prosperity means that under the guarantee of the socialism system with Chinese conditions, all the people will jointly create an increasingly developed and world-leading level of productive forces and enjoy an increasingly happy and better life [8]. Common prosperity is universal prosperity on the basis of eliminating polarization and poverty. It is the essential stipulation and goal of socialism and also one of the important contents of Deng Xiaoping’s theory of building socialism under Chinese conditions [9]. The "14th Five-year Plan for the Development of National Undertakings for the Aged and The Elderly Service System" further proposed to implement the national strategy to actively respond to the aging of the population, so that the elderly can share the fruits of reform and development and enjoy a happy old age [10]. The sustainability of sharing is more urgent in China, where aging is hitting our social security fund and will have a greater impact in the foreseeable future. There is a low matching degree between the taxpayer group and the social welfare benefit group, and the taxpayer group faces the problem of insufficient incentives [11]. Perfect old-age security system in a certain extent, has an important guarantee and promotion role on common prosperity, which can improve the quality of the laborers, resolve social contradictions, maintain social justice, effectively resolve social risks of market competition, and also have a certain function on adjusting the income gap, so as to create favorable conditions to achieve common prosperity. distribution is the core to promote common prosperity, while the UEBEI has a significant effect on income redistribution, and its income adjustment effect is positively correlated with the overall planning level and strength of the system [12]. Meanwhile, the improvement and construction of people’s livelihood security, including old-age insurance, is the foundation.

In order to promote the common prosperity of all the people, it’s crucial to solve the pension problem. The retired people have made contributions to the national construction, so they should share the fruits of economic development. To promote the high-quality development of pension service and respond to the expectation of the elderly people for a better life is also the inevitable proposition of The Times on the way to achieve the goal of common prosperity [13]. According to the data released by the Ministry of Human Resources and Social Security, the monthly pension of insured urban workers would be around 3,350 RMB in 2020, but many retired people only received around 2,000 RMB, and even some of them only received over 1,000 RMB. The gradual realization of common prosperity needs to promote more low-income groups to middle-income ranks, and the pension needs to be adjusted timely with the increase of people’s income, which provides an environment for the pension to rise continuously. According to the results of the seventh national census, the number of people aged 60 and above in China is 264.02 million, accounting for 18.70 percent of the total population, among which, the number of people aged 65 and above is 190.64 million, accounting for 13.50 percent. By the end of 2020, the urban old people aged 65 and above totaled 100.28 million, accounting for 11.14 percent of the total urban population (899.99million). Under the background of the population aging in China, about "who to support" "where to retire" "how much to spend on retirement" and a series of questions about the funds expenditure, all need to find a more suitable answer, the BEI fund spending system also need to be improved and optimized, as to better safeguard the common prosperity of aging society [14].

### 2.2 The influencing factors and indexes of UEBEI expenditure efficiency

Many factors will affect the expenditure efficiency of the basic endowment insurance fund for urban workers. Reducing the regional gap and pooling the BEI fund across the country will help to promote social equity, and improve well-being and happiness. Li Q et al. used Theil index, exploratory spatial data analysis and geographic detector to study regional differences and influencing mechanisms of UEBEI funds in China. Studies have shown that the total balance of UEBEI exists significant difference on the space distribution, deposit amount and institutional support rate has great influence to regional differences. It’s needed to insist on regional economic and social harmonious development, consolidate capture expends base, promote the national pooling of BEI funds formed gradually [15]. Cao Y & Liu JY discussed the impact of delayed retirement on pension wealth of urban workers from the perspective of actuarial neutrality based on the UEBEI in China. It is found that delayed retirement does not necessarily reduce the pension wealth of urban workers, and different retirement ages and genders have different requirements for actuarial neutral adjustment factors, and parameter variables have different incentive effects on delayed retirement. Therefore, it is suggested to strengthen actuarial neutral adjustment of retirement benefits, gradually implement delayed retirement policy, and establish promotion incentive mechanism of delayed retirement age policy implementation [16]. Abruquah, LA et al. studied the role of pension plan, self-reliance and intergenerational assistance related to China’s urban pension, and investigated the impact of pension reform and inequality among population and social groups on the life satisfaction of urban retirees. It is found that the self-reliance and pension benefits of the elderly are more effective in improving the life satisfaction of the urban retirees in China. It is suggested that the government formulate policies to promote the personal finance of the old people and improve the pension system to reduce the pension inequality [17].

Sustainable development is an urgent problem to be solved in China’s BEI system. Promoting national pooling and transferring the responsibility of collection from social security department to tax department are policies and measures to ensure the sustainable development of the BEI system. Xie, YT et al. explored the impact of the new birth policy on the actuarial balance of the UEBEI fund in China, constructed an actuarial balance model, and introduced a random mortality model to deal with longevity risk. The research shows that the increase of wage growth rate, the increase of fund investment income, the delay of retirement time and the increase of fund collection rate are all conducive to the sustainability of the UEBEI fund [18]. Chen, PJ & Shang, LH based on the background of national pooling and collection liability transformation, studies whether lowering contribution rate will affect the sustainability of China’s BEI fund, which is a policy issue in the reform of China’s BEI system. By constructing an improved theoretical actuarial model, they discuss the solvency of the national pooling account fund of BEI when the enterprise contribution rate drops to 16%. The conclusion shows that under the national pooling mode of stripping out historical debts and personal accounts, even if the contribution rate is reduced to 16%, China’s BEI fund will still have strong solvency in the long run [19].

### 2.3 Efficiency evaluation of endowment insurance based on DEA

Earlier scholars mainly used the traditional DEA model to study the efficiency of endowment insurance. Garcia MTM used DEA method to evaluate the efficiency of Portuguese pension fund management companies and ranked them according to the total productivity changes during 1994–2007. DEA-Malmquist index is used to estimate the change in total productivity which been decomposed into technically efficient change and technological change, and to identify best practices that will lead to improved performance in the market [20]. Laura Andreu et al. overcomes the possible limitations of the simple DEA method in the reference points of the efficient frontier may not be adequate. Based on the DEA-SBM model, the efficiency of strategic asset allocation, one of the most relevant decisions in pension fund management, is evaluated. The results reject the notion of a positive relationship between management resources and efficiency of strategic investment style, as well as highlights the relevance of SBM Variation III [21]. Ma, YR et al. made use of the statistical data of pension service institutions in 31 provinces of China from 2009 to 2014 to comprehensively evaluate the service efficiency of China’s pension service institutions with DEA method, and made an in-depth analysis of the dynamic changes and temporal and spatial characteristics of efficiency. It is found that due to low pure technical efficiency or low scale efficiency, the service efficiency of pension service institutions in most provinces of China is low, and there are different degrees of input redundancy and output insufficiency. The service efficiency of pension service institutions is unbalanced in regional development, and the service efficiency in East China is significantly higher than that in West China [22]. Toma, GC et al. used several indicators to measure the adequacy and participation of labor market in 26 Eu countries during 2007–2015, and analyzed and measured the performance and efficiency of pension insurance system in EU countries by using radar chart and DEA Malmquist method [23]. Lin SW et al. adopted the method of Additive Network DEA to study how the Public Service Pension Fund Management Board (PSPFMB) made the entrusting decision of selecting investment trust corporations (ITCs). By constructing a comprehensive prediction model, a more comprehensive performance measure is designed to measure the efficiency scores of 34 ITCs, including operating performance, equity fund performance, and bond fund performance, so as to ensure the long-term sustainability of pension funds [24].

In recent years, many scholars have begun to use the three-stage DEA model to study the operating efficiency of old-age social insurance. Hu YM et al. evaluated the efficiency of social security expenditure in China by using the three-stage DEA model based on panel data of Chinese provinces from 2007 to 2016. It is found that China’s social security expenditure has been increasing in the past ten years, and the efficiency of social security expenditure varies significantly among regions. The efficiency level of 29 provinces (cities) has not reached the efficiency frontier. Environmental factors and statistical noise have a significant impact on the efficiency of social security expenditure. If environmental factors and statistical noise are not considered, the efficiency of social security expenditure in China is likely to be underestimated [6]. Li ZG et al. measured the operating efficiency of basic pension insurance in 31 Provinces of China from 2014 to 2019 based on the three-stage DEA model. It is found that the operating efficiency of basic pension insurance in China is generally at a high level, but there is still room for improvement. There are significant regional differences in the operating efficiency of basic pension insurance in China. GDP, urbanization level and government public expenditure scale have a positive impact on the operating efficiency of basic pension insurance in the region, while the old-age dependency ratio has a significant negative impact [3]. Zhu H used the three-stage DEA model to evaluate and analyze the fiscal expenditure efficiency of basic public pension services in 16 districts of Shanghai, and found that among environmental factors, aging level and government power have a significant positive impact on expenditure slacks, while population size has no significant impact. Furthermore, it puts forward countermeasures such as attaching importance to government intervention ability and optimizing performance assessment index system [25]. Xue ZX & Ma ZP constructed a performance evaluation model of basic endowment insurance system based on the three-stage super-efficient SBM-DEA model. Taking the basic endowment insurance of employees as the research object, this paper analyzes the basic endowment insurance data of 31 Provinces in China from 2016 to 2020, and carries out experiments. The results show that the overall performance of the basic endowment insurance system for urban workers in all provinces of China shows a downward trend, and the development level of each province is obviously unbalanced. The regional characteristics are obvious, which showing a pattern of high in the east and low in the west, and the development level is not coordinated. In view of this phenomenon, the author uses Dagum to measure the spatial difference of performance of basic endowment insurance system, and predicts its dynamic evolution trend from time dimension by kernel function [26].

To sum up, academic studies on the performance of endowment insurance expenditure are mainly focused on fund sustainability, regional differences analysis, performance evaluation of endowment insurance system, and performance evaluation of BEI for urban and rural residents. There are few studies specifically on the efficiency evaluation of endowment insurance fund expenditure for urban workers. In particular, there are fewer studies using three-stage DEA method to evaluate the performance of UEBEI expenditure. With the deepening of urbanization and aging, the study on the performance evaluation of the UEBEI expenditure can also provide reference for the research on the BEI expenditure of rural residents, thus providing more research support for narrowing the urban-rural gap and realizing common prosperity. Therefore, this paper uses the three-stage DEA method that can effectively remove environmental factors and random errors, in order to estimate the more real efficiency level of the UEBEI in 31 provinces in China, so as to measure its management value and provide corresponding policy suggestions for further improving the BEI system in China. This article attempts to answer the following three questions:

1. Question 1: What is the expenditure efficiency of the UEBEI in China?
2. Question 2: How is the impact of environmental variables on the expenditure efficiency of the UEBEI in China?
3. Question 3: What about the regional differences of the UEBEI in China and how to narrow it?

We have three contributions in this paper as following, which not only has theoretical and academic value, but also has certain practical enlightenment for policy-making of relative decision departments. First of all, although many scholars have conducted abundant research on the endowment insurance, the overall research on the UEBEI in China is very few. Especially the lack of research on the expenditure efficiency of UEBEI. Secondly, this study applies the three-stage DEA model to evaluating the efficiency of UEBEI in China, which enriches the previous study about operating efficiency evaluation of endowment insurance. Thirdly, this paper also studies some external environmental factors to explore ways to improve its efficiency, and compares the efficiency differences in three regions of China, then some suggestions were given to improve the BEI system.

The writing framework of this paper is organized as follows. In the first part, background introduction, which defined the notion of BEBEI and its current situations in China; Literature review and research questions are included in part two, in which we review the relationship of BEI fund and common prosperity, as well as the related literatures on evaluation of endowment insurance by DEA method; The third part is the method introduction of three-stage DEA model, and the fourth part is the selection of indicators and data sources, including the introduction of input indicators and output indicators and environmental variables; The fifth part is an empirical study on the evaluation of expenditure efficiency of UEBEI fund by using three-stage DEA model. Finally, this paper summarizes and puts forward the policy enlightenment.

## 3. Methodology

As an evaluation tool to evaluate the relative efficiency of a group of decision-making units with multiple inputs and outputs, DEA model was first proposed by Charnes A et al in 1978, and the first DEA model was named CCR model [27]. Fried et al pointed out that the traditional DEA model did not consider the impact of environmental effects and random disturbances in efficiency measurement, and all decision-making units should be subjected to the same external conditions and random shocks, and adjusted inputs (or outputs) should be used to measure again to get the real efficiency of decision-making units, which is called three-stage DEA model [28]. The core idea of the method is to divide the factors that influence efficiency of input or output into the management level, environmental factors and random factors on the basis of traditional DEA method. Through the analysis and eliminating the influence of environmental factors and random factors, it can reevaluate by the adjusted model, so that the results reflected level of efficiency which only formed by the technical factors. It can make the evaluation result more real and accurate [29]. Therefore, this paper adopts the three-stage DEA method to study the expenditure efficiency of the UEBEI in 31 provinces and autonomous regions of China.

### 3.1 Stage 1: Traditional DEA model (BCC model)

In the first stage of DEA-BCC model, The original input-output data of the UEBEI are used to evaluate the initial efficiency. For any decision unit, the input-oriented duality BCC model can be expressed as:

$$\begin{array}{l} \;\min\theta -\varepsilon ({\hat{e}}^{T}S^{-}+e^{T}S^{+})\\ s.t.\{\begin{array}{l} \sum_{j=1}^{n}X_{j}\lambda_{j}+S^{-}=\theta X_{0}\\ \sum_{j=1}^{n}Y_{j}\lambda_{j}-S^{+}=Y_{0}\\ \lambda_{j}⩾0,S^{-},S^{+}⩾0 \end{array} \end{array}$$ (1)

Where j = 1, 2,…,n represents decision-making unit, and *λj* represents the weight of the *jth* decision-making unit. *X* and *Y* respectively represent the input and output combinations related to the expenditure efficiency of the UEBEI fund. *θ* represents the effective value of the decision- making unit, while *S+*, *S−* and *e* represent input slack variables, residual variables and non-Archimedean infinitesimals, respectively. If *θ* = 1,*S+* = *S−* = 0, the decision-making unit DEA is valid; If *θ* = 1,*S+*≠0,or *S−*≠0, the DMU is weak and effective; If *θ<*1, then the DMU is not DEA effective. The value of efficiency that calculated by BCC model is the technical efficiency (TE), which could be divided into pure technical efficiency (PTE), and scale efficiency (SE), that is, TE = SE*PTE. Fried believes that DMU performance is affected by management inefficiency, environmental factors and statistical noise, so it is necessary to separate these three influences.

### 3.2 Stage 2: Panel SFA (Stochastic frontier analysis) model

In the second stage, SFA regression was used to eliminate environmental factors and statistical noise, and the slack variables in the first stage were decomposed into three effects: environmental factors, management inefficiency and statistical noise. According to Fried et al (2002), the following regression model is constructed:

$$S_{nj}=f(Z_{j};\beta_{n})+v_{\mathrm{nj}}+\mu_{\mathrm{nj}};j=1,2,\cdots ,J;n=1,2,\cdots ,N$$ (2)

Where *Snj* represents the slack value of the *n*_*th*_ input of the *jth* decision-making unit, and *Zj* represents the environmental variable. The coefficient of the environmental variable is *βn*, the mixed error term is *Vnj+μnj*, and the random interference is *Vnj* and managerial inefficiency is *μnj*. The random error term *ν* ~*N*(0,σ*v*2) can be represented as the influence of random interference factors on the input slack variable. Managerial inefficiency *μ* is the influence of factors on the input slack variable, and it is assumed that *μ* ~*N+*(0,σ*μ*2) is expressed as a normal distribution function truncated at zero.

The purpose of SFA regression is to eliminate the influence of environmental factors and random factors on efficiency measurement, so that all DMUs can be adjusted into the same external environment. The adjustment formula is as follows:

$$X_{nj}^{A}=X_{nj}+[\max\left(f(Z_{j};{\hat{\beta }}_{n})\right)-f(Z_{j};{\hat{\beta }}_{n})]+[\max\left(v_{nj}\right)-v_{nj}]\;j=1,2,\ldots ,J;n=1,2,\ldots ,N$$ (3)

Where, $X_{nj}^{A}$ is the adjusted input; *X*_*nj*_ is input before adjustment; $\left[\max\left(f(Z_{j};{\hat{\beta }}_{n})\right)-f(Z_{j};{\hat{\beta }}_{n})\right]$ is found to be mediated by external environmental factors. $\left[\max\left(v_{nj}\right)-v_{nj}\right]$ means putting all decision units under the same luck level.

### 3.3 Stage 3: Adjusted traditional DEA model (BCC model)

In the third stage, the adjusted input and output index data of the UEBEI expenditure fund are replaced back into the original DEA model to recalculate the Efficiency of each DMUs. The efficiency value obtained is Comprehensive Efficiency after removing the influence of environmental factors and random factors, which can better reflect the relatively accurate and true efficiency level of the DMU.

## 4. Indicator selection and data source

### 4.1 Input and output indicators

The evaluation index system should be established on the basis of the principles of scientificity, systematicness, consistency, independence, feasibility, and comparability. Too many indicators will lead to information duplication and mutual interference, while not comprehensive indicators will lack sufficient representativeness. In addition, full consideration must be given to the overlap between indicators to prevent bias and misleading results as designing indicators [3]. An efficient and sustainable pension system should have sufficient financial solvency to avoid spending more than it earns. In the analysis of the expenditure efficiency of UEBEI fund in China, the selection of input and output indicators is the key to evaluation, and different combinations of factors often produce certain differences in the output of the results [30]. In order to ensure the accuracy of the calculation of the expenditure efficiency of urban employee’s basic endowment insurance fund, we found through literature review that the input indicators mainly include fund income, the growth rate of wage, and the number of people insured etc. Output indicators mainly include fund expenditure, number of recipients, pension substitution rate and accumulated fund balance etc. Based on previous research results and the actual situation of UEBEI fund, this paper finally selects fund income and the number of insured persons as input indicators, and fund expenditure and accumulated fund balance as output indicators [3, 6, 31–33] (see Table 1).

**Table 1:** Descriptive statistical analysis of input, output and environmental variables.

Category	Indicators	Mean	Sd.	Min	Median	Max
**Inputs**	UEBEI Fund Income (100 Million RMB)	1457.10	998.28	79.50	1145.20	5593.20
	Number of insured persons (10 thousand people)	1347.91	1055.54	21.10	1074.30	5392.40
**Outputs**	UEBEI Fund Expenditure (100 Million RMB)	1381.95	904.86	51.80	1168.80	3761.50
	Fund accumulated balance (100 Million RMB)	1522.55	2096.51	-557.20	834.80	12343.60
**Environmental variables**	Fiscal autonomy %	45.37	19.24	2.63	42.31	92.59
	The level of urbanization %	61.40	11.62	31.57	59.88	89.30
	The old-age dependency ratio %	16.12	3.93	7.01	15.88	25.48
	The level of marketization %	51.64	8.00	39.60	50.80	83.90

### 4.2 The environment variable

Environmental variables refer to those factors that, apart from input and output variables, do have an impact on efficiency but are not subject to the subjective control of the sample and cannot be changed in a short time. That is to say, environmental variables have an impact on the expenditure efficiency of urban employee’s basic endowment insurance fund in China to a certain extent, but cannot have a subjective control effect on the sample. The selection of environmental variables based on separation hypothesis is crucial to the three-stage DEA. Considering the characteristics of urban employee’s basic endowment insurance fund in China and the research results of previous scholars [3, 6, 26], this article takes the fiscal autonomy, urbanization level, the dependency ratio of the elderly population, and the level of marketization as environmental variables that affect urban employee’s basic pension insurance fund in China.

Fiscal autonomy. It is a system whereby local governments or ethnic autonomous areas independently decide and manage local financial affairs according to law within the framework defined by the constitution and financial law of a country. In this paper, the proportion of local financial revenue in financial expenditure is used to measure it. Since the reform of the tax system, the Chinese government has centralized financial power and decentralized administrative power. Subsidies income has been a main component of the BEI fund income, to a certain extent, affects the scale and efficiency of the UEBEI’s fiscal expenditure, and the change of the local public finance expenditure level is related to the total amount of the UEBEI fund.

The level of urbanization. Level of Urbanization refers to the urbanization degree of a region, which is an important indicator of regional economic development, and is usually expressed as the percentage of the urban population to the total population. The index is used to reflect the process and aggregation degree that population gathered to the city, the process of urbanization is often accompanied by urban agglomeration of the capital and labor in each region, the agglomeration effect will promote local employment and economic development, improve the level of income, improve the living environment and the level of medical & education, provide better material conditions for local financial subsidies and individual pay cost, The scale effect formed by population agglomeration also helps to reduce the regulatory cost of providing public services by the government in the region.

The old-age dependency ratio. We used the old-age dependency ratio to measure the impact of aging on the expenditure efficiency of UEBEI fund, which usually expressed as a percentage ratio of the number of old-age people in a population to the number of working-age people. The dependency ratio is one of the indicators reflecting the social consequences of population aging from an economic perspective, indicating how many old-age people should be supported by every 100 working-age people. The public’s old-age service needs are closely related to various demographic factors, and meeting the public’s needs is the starting point and end point for the government to provide public services. The demographic structure will have a certain impact on the expenditure efficiency of the UEBEI system. As the continuous rising of China’s aging degree, the old-age dependency ratio also improves correspondingly, the aging population then have greater demand for the basic endowment insurance, and more money will be needed to guarantee, thus affect the expenditure efficiency of the UEBEI in China.

The level of marketization. This indicator refers to the level and degree of marketization development in a certain region, which reflects the comprehensiveness of the legal environment and maturity of the factor market, and is measured by the proportion of tertiary industry in this paper. The proportion of the added value of the tertiary industry in the GDP of a region is an important statistical index, which reflects the stage of economic development of a country or region and the quality of people’s living standards. The expenditure of basic endowment insurance fund is related with the level of regional macro-economic development, and the level of regional economic development has a great relationship with the performance of government public service. Marketization promotes the demand for labor and thus facilitates social insurance coverage, and normally a higher level of marketization signifies a higher efficiency of capital allocation and fiscal expenditure [6]. It is generally believed that the stronger the regional economic strength is, the better the basic conditions will be, and the higher the level of social security including old-age security will be.

### 4.3 The data source

This study takes the UEBEI data on 31 provinces and municipalities of China from 2016 to 2020. The data of input, output indicators and environmental variables are derived from China Statistical Yearbook and provincial statistical yearbook in 2021.

## 5. Empirical analysis on expenditure efficiency of the UEBEI in China

Since we use the three-stage DEA model for analysis, it is necessary to conduct correlation tests on input indicators and output indicators to determine whether they are positively correlated with each other. That is, an increase in the input variables cannot cause a decrease in the output variables. From the analyzed results of the estimated correlation coefficients by SPSS 23.0, the correlation coefficients between all input indicators and output indicators are positive and greater than 0.56. The two are significantly correlated at the 0.01 significance level, indicating that the input index has a high correlation with the output index, indicating that the selected variables are appropriate and meets the data requirement of DEA model. The Pearson correlation matrix of input-output data indicators is shown in Table 2.

**Table 2:** Correlation analysis of input index and output index.

	UEBEI Fund Expenditure (100 Million RMB)	Number of insured persons (10 thousand people)	UEBEI Fund Income (100 Million RMB)	Fund accumulated balance (100 Million RMB)
**UEBEI Fund Expenditure (100 Million RMB)**	1			
**Number of insured persons (10 thousand people)**	0.904***	1		
**UEBEI Fund Income (100 Million RMB)**	0.921***	0.832***	1	
**Fund accumulated balance (100 Million RMB)**	0.787***	0.816***	0.561***	1

Note: ***. P< 0.01

### 5.1 Stage 1: DEA model analysis

According to the original data of input-output indicators and input-oriented BCC model, this paper uses DEAP2.1 software to calculate the expenditure efficiency of the UEBEI in China from 2016 to 2020. Table 3 shows the average expenditure efficiency of the UEBEI in each province and the annual comprehensive technical efficiency status in the sample year. From the calculation results, the following results can be obtained without considering external environmental factors and random error: (1) Generally, the UEBEI in China has a good expenditure efficiency in the sample years, with the average value of TE, PTE and SE being 0.739, 0.789 and 0.940 respectively. As can be seen from Table 3, the technical efficiency of some provinces, such as Zhejiang and Shanghai, reached 1.000 in 2020, that is, they are highly efficient but not at the forefront of technical efficiency. (2) From the regional perspective, the average technical efficiency of East, Central and West China is 0.725, 0.729 and 0.760, respectively, showing a trend of West China > Central China> East China. The average technical efficiency of East and Central China has not reached to the national average level.

**Table 3:** Comprehensive technical efficiency of China’s UEBEI from 2016 to 2020.

Region	Province	Technical Efficiency (TE)					
		2016	2017	2018	2019	2020	Mean
**East China**	Beijing	0.547	0.554	0.537	0.556	0.778	0.590
	Tianjin	0.730	0.682	0.779	0.748	0.873	0.736
	Hebei	0.739	0.692	0.679	0.761	0.805	0.708
	Shanghai	0.703	0.811	0.803	0.845	1	0.831
	Jiangsu	0.692	0.669	0.647	0.676	0.866	0.706
	Zhejiang	0.698	0.649	0.710	0.755	1	0.755
	Fujian	0.672	0.666	0.641	0.652	0.832	0.691
	Shandong	0.688	0.747	0.689	0.730	0.871	0.745
	Guangdong	0.579	0.544	0.517	0.587	0.777	0.599
	Hainan	0.826	0.741	0.699	0.737	0.938	0.780
	Liaoning	0.800	0.809	0.791	0.810	0.976	0.836
	**Mean**	**0.698**	**0.688**	**0.681**	**0.681**	**0.714**	**0.725**
**Central China**	Shanxi	0.771	0.698	0.718	0.731	0.872	0.749
	Jilin	0.782	0.727	0.642	0.775	0.913	0.766
	Heilongjiang	0.905	0.845	0.751	0.824	0.939	0.842
	Anhui	0.677	0.642	0.746	0.662	0.793	0.677
	Jiangxi	0.725	0.654	0.633	0.756	0.815	0.714
	Henan	0.709	0.696	0.676	0.667	0.807	0.709
	Hubei	0.737	0.718	0.706	0.703	0.770	0.711
	Hunan	0.701	0.677	0.592	0.681	0.737	0.662
	**Mean**	**0.751**	**0.707**	**0.683**	**0.725**	**0.831**	**0.729**
**West China**	Gansu	0.815	0.779	0.719	0.763	0.889	0.792
	Guangxi	0.723	0.659	0.703	0.695	0.815	0.704
	Guizhou	0.779	0.677	0.627	0.681	0.806	0.703
	Inner Mongolia	0.774	0.627	0.757	0.831	0.886	0.762
	Ningxia	0.827	0.811	0.786	0.852	0.915	0.839
	Qinghai	0.946	0.891	0.856	1	0.932	0.886
	Tibet	1	0.845	1	0.965	1	0.943
	Xinjiang	0.746	0.720	0.689	0.723	0.759	0.725
	Chongqing	0.697	0.724	0.674	0.710	0.776	0.708
	Sichuan	0.697	0.522	0.663	0.750	0.849	0.688
	Yunnan	0.627	0.782	0.638	0.652	0.789	0.672
	Shaanxi	0.734	0.663	0.646	0.683	0.748	0.692
	**Mean**	**0.780**	**0.725**	**0.730**	**0.775**	**0.847**	**0.760**
**Mean of all provinces**		**0.743**	**0.707**	**0.700**	**0.741**	**0.856**	**0.739**

The calculation results in Table 3 do not consider the impact of environmental factors and random factors and do not truly reflect the actual situation of UEBEI in China. Due to the developing differences in the level of aging in different regions, the external environment faced by the UEBEI fund in different regions also vary widely. Therefore, it is necessary to reevaluate the efficiency of China’s UEBEI fund after excluding the influence of environmental factors and random interference factors, so as to reflect the accurate level of expenditure efficiency.

### 5.2 Stage 2: SFA model analysis

At stage 2, the slack variable (UEBEI Fund Income, and the Number of UEBEI Insured Persons) obtained from stage 1 was introduced as the dependent variable, and fiscal autonomy, the level of urbanization, the old-age dependency ratio, and the level of marketization were introduced as independent variables in the model. After build the panel SFA regression model by using the Frontier 4.1 software, the Maximum Likelihood Estimation (MLE) is used to estimate the impact of environmental variables.

As shown in Table 4, the likelihood ratio(LR) test of the SFA model passed the significance test at the 1% level, and rejected the null hypothesis that there was no managerial inefficiency, indicating that it was reasonable to apply the SFA model in stage 2. Both σ2 and γ value passed the significance test (γ = 0.52;& γ = 0.68), indicating that compared with random error, managerial inefficiency in the mixed error term has a dominant influence on the slack variable. Moreover, the estimated coefficients of the four environmental variables also passed the significance test, indicating that environmental factors have a significant impact on the slack values of UEBEI Fund Income and the Number of UEBEI Insured Persons; therefore, applying the SFA model to separate the environmental variables and statistical noises is reasonable. Therefore, in order to further adjust the input variables, it is crucial to eliminate the influence of environmental factors on the redundancy of the input variables through SFA analysis. Since environmental variables are regressions in the input slack value; if the estimated coefficient of an environmental variable is negative, then increasing the environmental variable can reduce the input slack, which is beneficial for improving efficiency. If the estimated coefficient is positive, then increasing the environmental variable will increase the input redundancy, which is not conducive to improving efficiency. The findings, based on Table 4, are as follows:

**Table 4:** Results of the SFA model in Stage 2.

Variables	Slack of UEBEI Fund Income	Slack of The Number of UEBEI Insured Persons
**Constant**	307.71***	418.17***
**Fiscal autonomy %**	5.10***	5.24***
**The level of urbanization %**	-4.27**	-5.49
**The old-age dependency ratio %**	15.15***	21.96***
**The level of marketization %**	-7.23***	-10.16***
**σ2**	42,384.51***	94,066.06***
**γ**	0.52***	0.68***
**log likelihood**	-1,208.28***	-1,249.70***
**LR test**	28.57***	70.88***

Note: * p<0.1

** p<0.05

*** p<0.01,**Note:** indicates significant p values at the 10%, 5%, 1%.

#### 5.2.1 Fiscal autonomy

The regression results show that the regression coefficients of fiscal autonomy, slack of UEBEI fund income and slack of the number of UEBEI insured persons are all positive, and pass the significance test of 1%. It shows that the increase of fiscal autonomy is not conducive to the improvement of the efficiency of pension fund expenditure in China, which is controversial with our hypothesis and some previous researches. The reason may be normally when local governments have more financial autonomy and a larger team size, their efficiency of UEBEI increases. however, An imbalance between centralized financial power and decentralized administrative power is likely to lead to behavioral preferences for local governments and to aggravate competition among local governments, thereby affecting the operating efficiency of UEBEI. The increase of fiscal autonomy, that is, the proportion of fiscal revenue in fiscal expenditure is too large, and the level of public financial expenditure is low, which is not conducive to increasing the investment of the total scale of UEBEI and improving the efficiency of fund expenditure, and increases the fund management cost of each region. On the other hand, high fiscal autonomy is not beneficial to promoting the national pooling of BEI. Therefore, when fiscal autonomy passes a certain point, it will be unfavorable to improve the operating efficiency of the UEBEI.

#### 5.2.2 The level of urbanization

There is a negative correlation between the level of urbanization and the slack variables of fund income and the number of UEBEI insured persons in China(p<0.01). The agglomeration effect of the urbanization process has promoted local employment and economic development, also increase the fund income and relieve the pressure of local financial subsidies for old-age insurance. Moreover, rural migrants’ mentality has shifted to becoming more closely correlated with the mentality of urban employees, and the demand for old-age service is even stronger. The urbanization process increases the coverage of the endowment fund amount and is conducive to improving the expenditure efficiency of the UEBEI.

#### 5.2.3 The old-age dependency ratio

There is a positive correlation between the old-age dependency ratio and the slack variables of fund income and the number of UEBEI insured persons(p<0.01). The results show that the old-age dependency ratio is not conducive to improving the expenditure efficiency of the UEBEI, which is also contradicts the conclusions of most previous studies. With the urgency of deep aging in China, more people will pay attention to the pension system and promote the improvement of UEBEI coverage rate, and the government will also increase the corresponding financial input or subsidies. With the increase of the old-age dependency ratio, more and more retired employees will participant in receiving pension benefits, and the expenditure of UEBEI may become more efficient. However, with the further deepening of the aging degree, the elderly support has been rising faster in recent years, and the old-age population’s demand for BEI and the overall pension pressure of the society have increased significantly, which increases the operation pressure of the BEI fund. However, due to the current pension system starts late, and the pension system development imbalance at every level, thus causing the accumulation of the pension in China is very limited, there is a big gap in the size of total pension reserves of GDP compared with the developed countries. Lots of problems with aging need to be solved, such as the limited pension stock, incremental shortage, late overall planning, which may impede the expenditure efficiency of UEBEI.

#### 5.2.4 The level of marketization

The marketization level is negatively correlated with the slack variables of fund income and the number of UEBEI insured persons(p<0.01), which shows that the marketization level has significantly inhibited the fund investment redundancy. This means that the higher the level of regional economic development and the quality of people’s living standards, the more conducive to improving the expenditure efficiency of the UEBEI fund. The stronger the regional economic strength, the better the basic conditions, the proportion of the added value of the tertiary industry in the regional GDP will increase, the demand for labor force and the number of insured people will increase as well, then the level of endowment insurance will also be improved. When the fund investment is within the appropriate range and has not caused redundancy and waste yet, the expenditure efficiency of UEBEI will increases.

### 5.3 Stage 3: DEA-BCC model analysis

As seen from the result of stage 2, the environment variables and random error are correlated with the slack variables of fund income and the number of UEBEI insured persons in all provinces. Therefore, Therefore, it is necessary to adjust the original input variables so that all regions face the same environment and luck, and then investigate the real operation efficiency of the UEBEI. DEAP 2.1 was used to measure the efficiency of the adjusted input-output data using the input-oriented BCC-DEA model again, and the real efficiency of the UEBEI in each province in stage 3 was obtained, and the changes of efficiency values between the stage 1 and stage 3 were listed (as shown in Table 5). As can be seen from the calculation results in Table 2, after excluding the influence of environmental factors and random interference, the comparison shows that there is a certain deviation in the calculation results of the two stages, indicating that it is meaningful to build the SFA model for estimation in the second stage.

**Table 5:** Measurement results and comparison table of Stage 1 and Stage 3.

Region	Province	Results of Stage 1				Results of Stage 3				Improve Range		
		TE	PTE	SE	RS	TE	PTE	SE	RS	TE	PTE	SE
**East China**	Beijing	0.590	0.905	0.652	drs	0.892	0.975	0.915	irs	0.302	0.070	0.263
	Tianjin	0.736	0.736	1	-	0.609	0.98	0.621	irs	-0.127	0.244	-0.379
	Hebei	0.708	0.708	1	-	0.803	0.955	0.841	irs	0.095	0.247	-0.159
	Shanghai	0.831	0.946	0.879	drs	0.966	0.992	0.974	irs	0.135	0.046	0.095
	Jiangsu	0.706	0.778	0.907	drs	0.95	0.957	0.992	irs	0.244	0.179	0.085
	Zhejiang	0.755	0.812	0.929	drs	0.946	0.969	0.976	irs	0.191	0.157	0.047
	Fujian	0.691	0.717	0.964	drs	0.559	0.982	0.569	irs	-0.132	0.265	-0.395
	Shandong	0.745	0.765	0.973	drs	0.912	0.964	0.946	irs	0.167	0.199	-0.027
	Guangdong	0.599	0.821	0.730	drs	0.918	0.941	0.975	irs	0.319	0.120	0.245
	Hainan	0.780	0.780	1	-	0.306	0.993	0.308	irs	-0.474	0.213	-0.692
	Liaoning	0.836	0.839	0.997	irs	0.928	0.977	0.95	irs	0.092	0.138	-0.047
**Central China**	Shanxi	0.749	0.856	0.875	drs	0.71	1	0.71	irs	-0.039	0.144	-0.165
	Jilin	0.766	0.769	0.996	irs	0.618	0.975	0.634	irs	-0.148	0.206	-0.362
	Heilongjiang	0.842	0.855	0.986	irs	0.802	0.972	0.825	irs	-0.040	0.117	-0.161
	Anhui	0.677	0.749	0.904	drs	0.725	0.987	0.734	irs	0.048	0.238	-0.170
	Jiangxi	0.714	0.719	0.993	drs	0.621	0.977	0.635	irs	-0.093	0.258	-0.358
	Henan	0.709	0.709	1	-	0.763	0.957	0.798	irs	0.054	0.248	-0.202
	Hubei	0.711	0.711	1	-	0.814	0.945	0.861	irs	0.103	0.234	-0.139
	Hunan	0.662	0.704	0.941	drs	0.75	0.952	0.788	irs	0.088	0.248	-0.153
**West China**	Gansu	0.792	0.806	0.983	drs	0.448	0.985	0.455	irs	-0.344	0.179	-0.528
	Guangxi	0.704	0.714	0.987	drs	0.637	0.975	0.653	irs	-0.067	0.261	-0.334
	Guizhou	0.703	0.750	0.938	drs	0.498	0.987	0.505	irs	-0.205	0.237	-0.433
	Inner Mongolia	0.762	0.767	0.993	drs	0.627	0.978	0.641	irs	-0.135	0.211	-0.352
	Ningxia	0.839	0.839	1	-	0.301	0.993	0.304	irs	-0.538	0.154	-0.696
	Qinghai	0.886	0.920	0.963	irs	0.277	0.992	0.279	irs	-0.609	0.072	-0.684
	Tibet	0.943	0.945	0.998	drs	0.273	0.997	0.274	irs	-0.670	0.052	-0.724
	Xinjiang	0.725	0.877	0.826	drs	0.65	0.988	0.658	irs	-0.075	0.111	-0.168
	Chongqing	0.708	0.735	0.964	drs	0.688	0.979	0.703	irs	-0.020	0.244	-0.261
	Sichuan	0.688	0.755	0.912	drs	0.946	0.948	0.998	irs	0.258	0.193	0.086
	Yunnan	0.672	0.787	0.854	drs	0.591	0.998	0.592	irs	-0.081	0.211	-0.262
	Shaanxi	0.692	0.697	0.993	drs	0.641	0.971	0.66	irs	-0.051	0.274	-0.333
Mean of all Provinces		0.739	0.789	0.940		0.683	0.976	0.702		-0.057	0.186	-0.238

**Abbreviations:** TE, technical efficiency; PTE, pure technical efficiency; SE, scale efficiency; RS, return to scale; irs, returns to scale increased; drs, returns to scale decreased; -, represent returns to scale unchanged.

On a whole, the mean of TE, PTE, and SE of UEBEI in stage 3 are 0.683, 0.976 and 0.702, respectively. The TE is lower than that in stage 1, which is affected by both the improvement of PTE and the decline of SE. From the state of returns to scale presented in stage 1, six provinces including Tianjin, Hebei and Hainan etc. are in a state of returns to scale unchanged, and most provinces are in a state of returns to scale decreased. Only Liaoning, Jilin, Heilongjiang and Qinghai provinces are in a state of returns to scale increased, indicating that the four provinces will have a positive effect on the improvement of the UEBEI’s fund efficiency if continuing to increase the investment. Contrast to stage 1, in addition to the four provinces of Liaoning, Jilin, Heilongjiang and Qinghai, the rest of the provinces in stage 3 is at a state of returns to scale increased, indicating that the scale of UEBEI in these provinces has yet to reach its technical level and management efficiency of the optimal size, and should be appropriately increased the fund investment of UEBEI.

#### 5.3.1 Overall efficiency analysis

**Fig 1:**
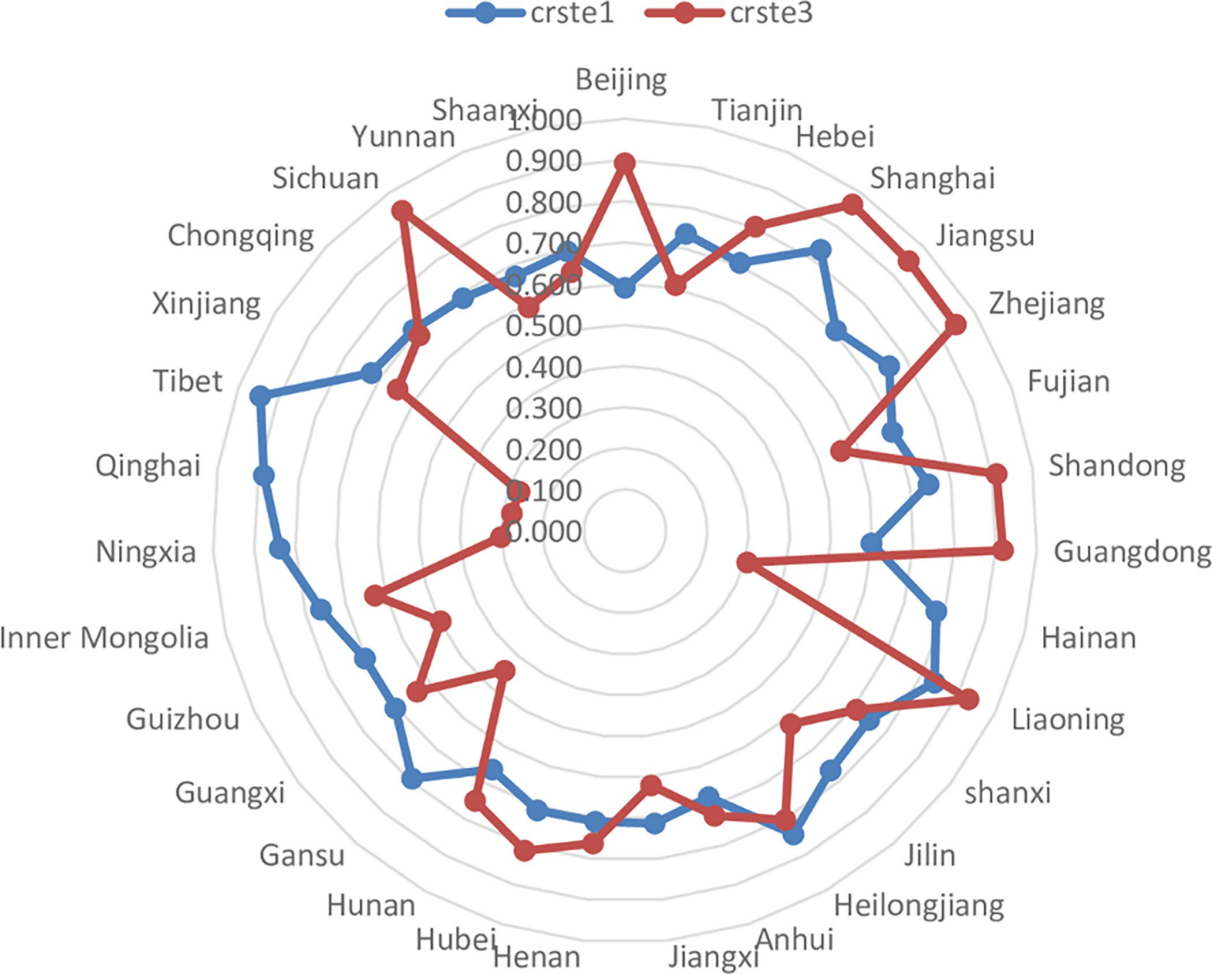
TE radar diagram of Stage 1 and Stage 3.

*(1) TE of the UEBEI*. The technical efficiency index is used to measure the overall efficiency of each province/municipality in the investment, use and capital management of the UEBEI. After excluding the external environment and random factors, the mean of TE in provinces and cities decreased from 0.739 to 0.683, down by 7.58%. As can be seen from Fig 1, the overall efficiency of 13 provinces, including Beijing, Hebei, Shanghai and Jiangsu etc., has been improved in stage 3. Guangdong province has the highest improvement rate of 53.26%, followed by Beijing, which has improved 51.19%. The overall efficiency of the other 18 provinces declined to varying degrees. Among them, Hainan, Ningxia, Qinghai and Xizang adjusted their overall TE, with low efficiency values of 0.306, 0.301, 0.277 and 0.273, respectively. In particular, Tibet decreased by 71.05%. Among the 31 provinces in stage 3, there is no province whose efficiency value is 1, that is, no one is at the forefront of efficiency. The provinces with relatively high efficiency are Shanghai and Jiangsu, whose TE value is higher than 0.95, while the provinces with TE value higher than 0.85 in stage 1 are Tibet and Qinghai whose efficiency value decreases significantly in stage 3. It shows that the efficiency value of these provinces in stage 1 may be related to the external environment and operating luck, and cannot reflect their real fund management level. It also shows that the stability of the expenditure efficiency of the UEBEI in all provinces fluctuates to a certain extent, and there are still great differences of efficiency in different regions. Since TE = PTE*SE, we will continue to analyze pure technical efficiency and scale efficiency.

**Fig 2:**
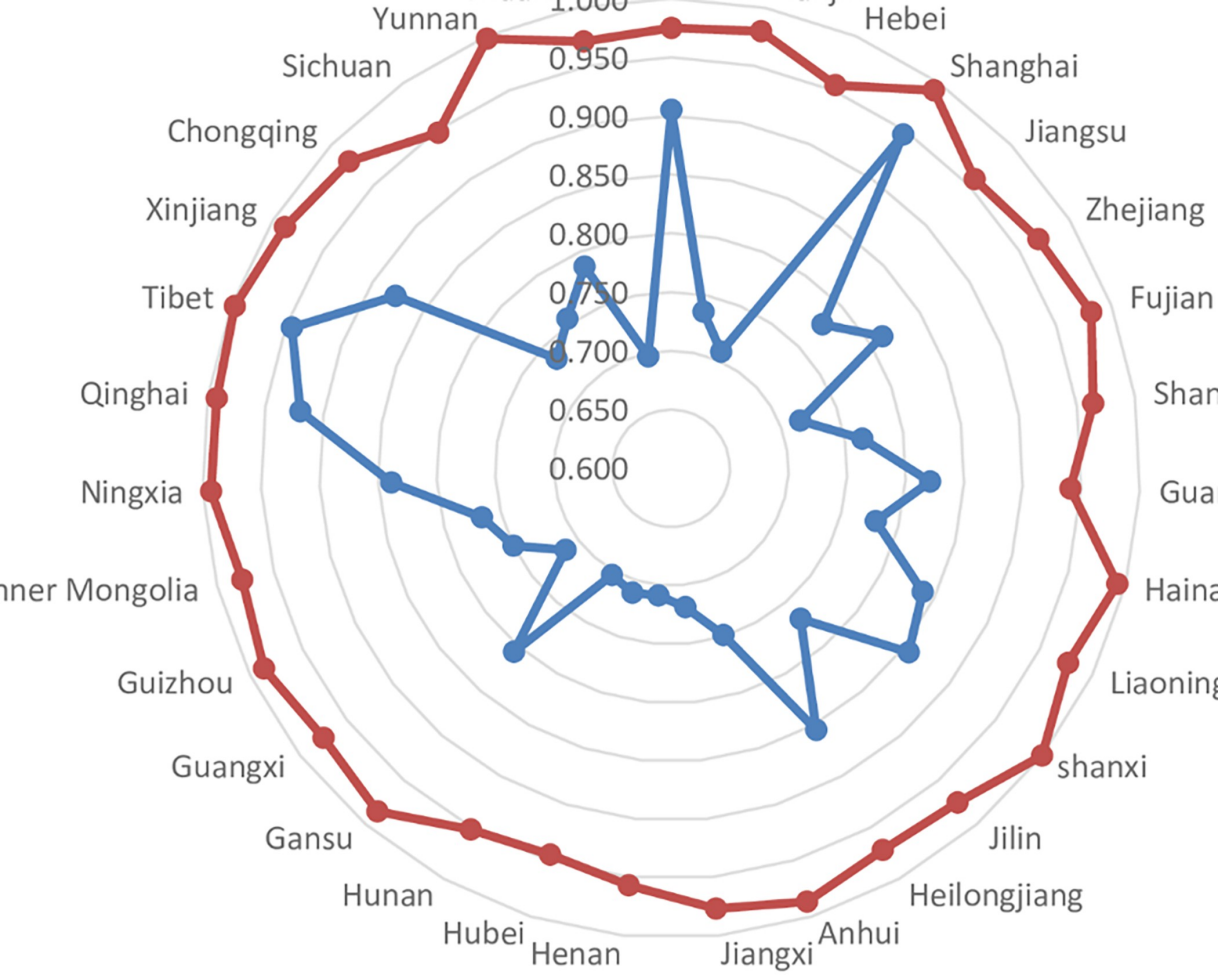
PTE radar diagram of Stage 1 and Stage 3.

*(2) PTE of the UEBEI*. The pure technical efficiency index reflects the distribution and management of the UEBEI in various provinces/municipalities. The overall PTE increased from 0.789 in stage 1 to 0.976 in stage 3, an increase of 23.58%. As shown in Fig 2, compared with the comprehensive technical efficiency, the number of provinces with high PTE increased significantly. A total of 18 provinces had efficiency values higher than the national average, among which the province with the most obvious improvement was Shaanxi province (39.31%), while Fujian, Jiangxi, Hunan and Guangxi all increased by more than 35%. Before the adjustment, no provinces realized technical efficiency; After adjustment, Shanxi Province has realized pure technical efficiency, which indicates that the UEBEI fund in Shanxi Province is at the current technical level, and the use of its investment resources is effective. The TE of other provinces still has some space for improvement. However, the PTE of 31 provinces after adjustment has been improved, and the efficiency value is all above 0.94, indicating that the technical level of management and allocation of the UEBEI fund is generally good.

**Fig 3:**
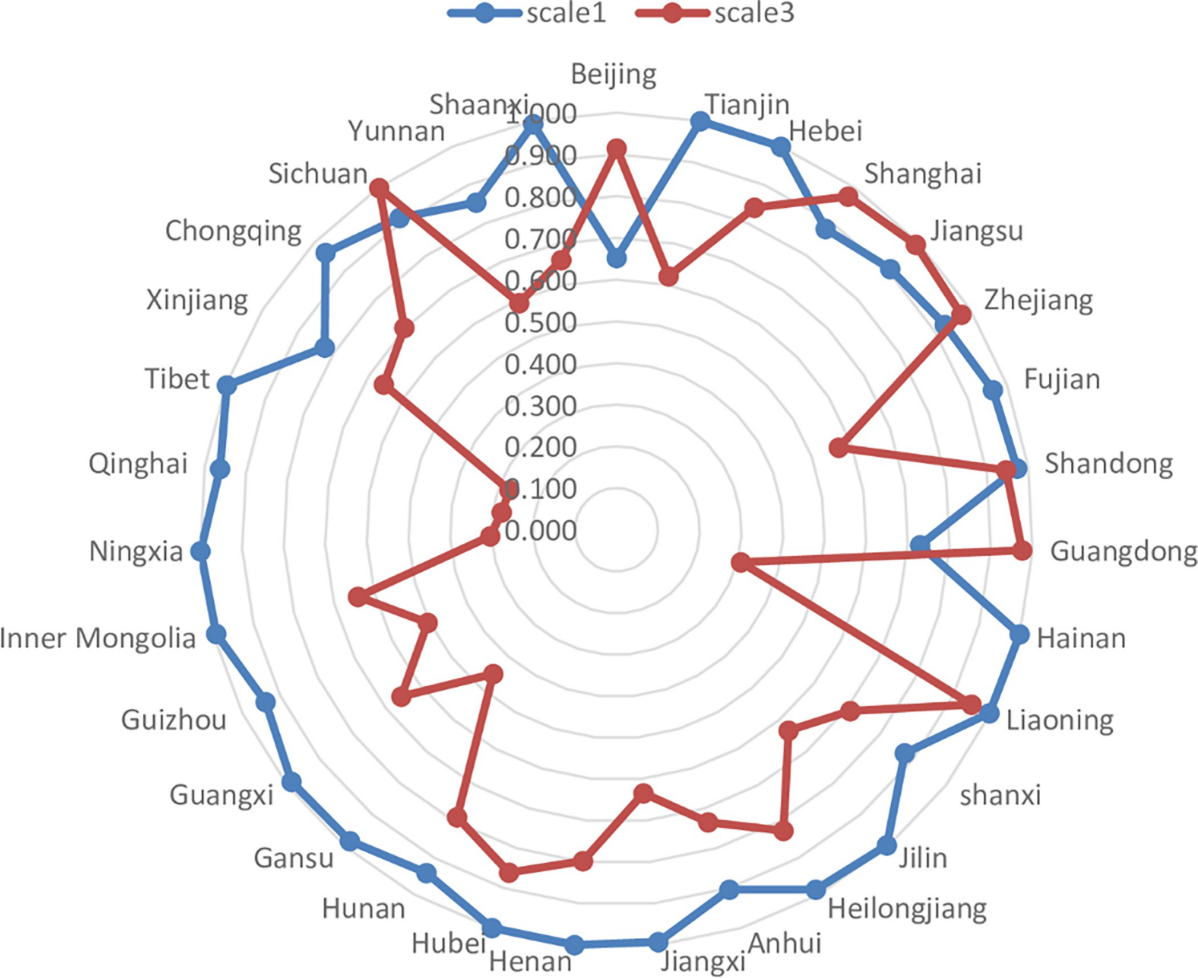
SE radar diagram of Stage 1 and Stage 3.

*(3) SE of the UEBEI*. The scale efficiency analysis reflects the gap between the current scale and the optimal scale of the UEBEI in all provinces and municipalities. After excluding external environment and random influencing factors, the change of scale efficiency in each province is shown in Fig 3. The overall average scale efficiency of UEBEI in 31 provinces dropped from 0.940 before the adjustment to 0.702 after the adjustment, a drop of 25.27%. The mean value of scale efficiency in most provinces showed a certain degree of decline, and the decline rate was higher than the national average in 15 provinces. Among them, the scale efficiency in Tibet and Qinghai decreased most obviously from 0.998 and 0.963 in stage 1 to 0.274 and 0.279 in stage 3, with a decline of 72.55% and 71.03%, respectively. As a result, the overall expenditure efficiency of the UEBEI in these provinces is relatively low. Even if they perform well in pure technical efficiency, they cannot make up for their lack of scale efficiency. The reason may be that the economic development of these provinces is relatively backward, which leads to the insufficient scale and low scale efficiency of the UEBEI. Therefore, these provinces should pay attention to rational allocation of resources and improve the efficiency of resource utilization. Only six provinces of Beijing, Shanghai, Jiangsu, Zhejiang, Guangdong, Sichuan, the scale efficiency in stage 3 increased. Among the provinces with biggest scale efficiency increase, Beijing’s efficiency has the most significant increase of 40.34%, up from 0.652 in stage 1 to 0.915 in stage 3. It shows the size of its capital is more reasonable, and there is not much difference with the optimal operation scale.

**Fig 4:**
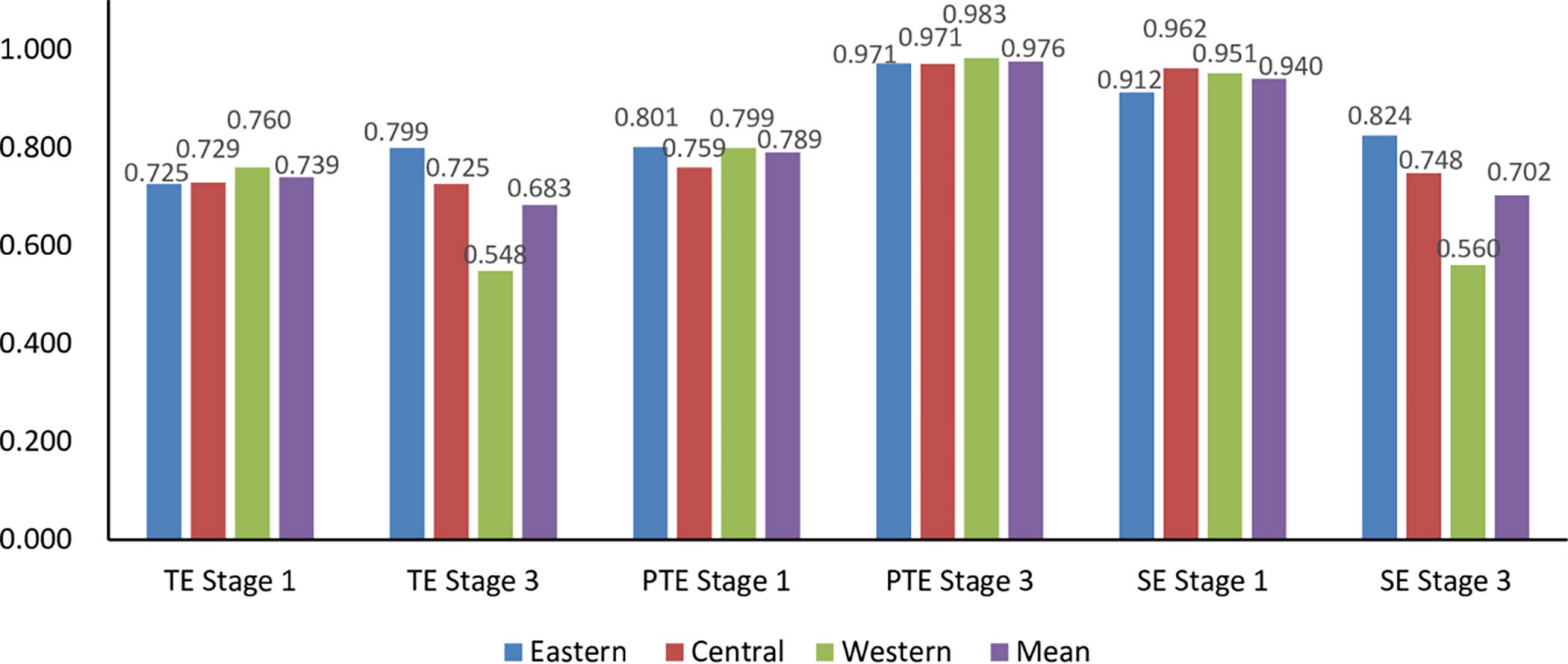
Comparison of the efficiency in different regions in Stage 1 and Stage 3.

**5.3.2 Regional distribution of TE of the UEBEI.** In order to further analyze the differences in the expenditure efficiency of the UEBEI in different regions of China, according to the level of economic development and geographical location of China, combined with the long-term evolution, this paper divides 31 provincial administrative units in China into three main economic zones: East China, Central China and West China. In terms of region, the average comprehensive technical efficiency of eastern, central and western regions changed from 0.725, 0.729 and 0.760 in stage 1 to 0.799, 0.725 and 0.548 in stage 3 respectively, and changed from the pattern of West China > Central China> East China to the overall pattern of East China > Central China > West China. After eliminating the influence of environmental factors and statistical noise, The efficiency of the eastern region increased by 10.21%, while that of the central and western regions decreased by 0.55% and 27.89%, respectively. As can be seen from Fig 4, the average decline of TE in the western region was the most obvious, followed by the central region. The reason is that the higher pure technical efficiency and scale efficiency in the eastern region ensure the leading comprehensive technical efficiency, which is matched with the economic strength and financial resources of the eastern region. The comparison of efficiency calculation results of the two stages shows that the investment of the UEBEI in West China has not reached the optimal scale. Further measures should be taken, such as strengthening policy publicity, increasing fund income, ensuring basic employment and so on, to further improve the endowment insurance system. In terms of the mean value of pure technical efficiency in different regions, the mean value of efficiency in East China, central and western regions increased from 0.801, 0.759 and 0.799 in stage 1 to 0.971, 0.971 and 0.983 in stage 3 respectively. The central region showed the most obvious improvement of 27.88%, higher than the national average. However, it is worth noting that the mean values of scale efficiency in the eastern, central and western regions decreased from 0.912, 0.962 and 0.951 in stage 1 to 0.824, 0.748 and 0.560 in stage 3 respectively. The scale efficiency in the western region showed the most obvious decline, with a decrease of 41.09%, which was far lower than the national average level. Low scale efficiency is still the main reason that restricts the improvement of scale efficiency in West China.

**Fig 5:**
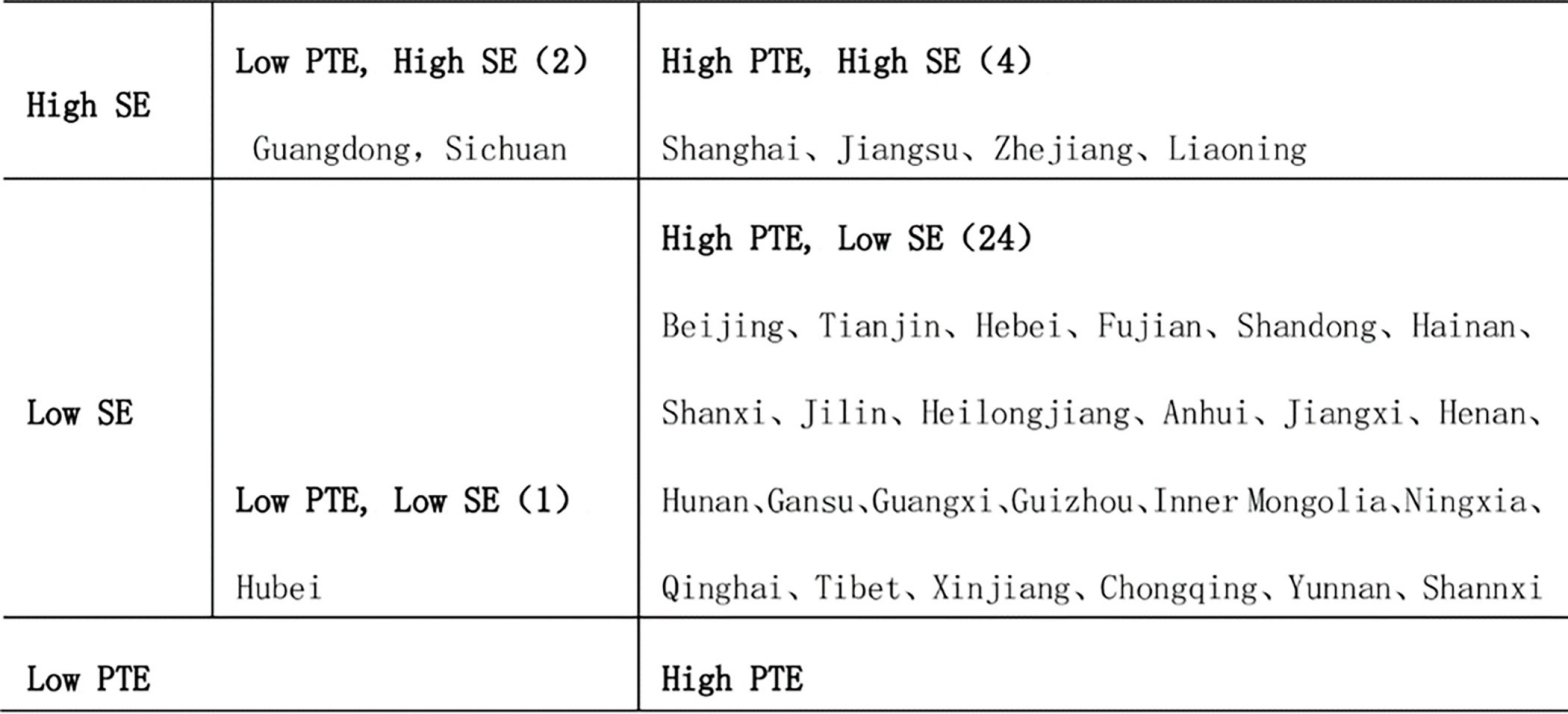
Efficiency distribution of each province in the Stage 3.

In order to further analyze the efficiency improvement strategy of each region, the efficiency level of each province is divided into corresponding types. According to the value of PTE and SE of each region, 0.95 is taken as the relative critical point, where more than 0.95 indicates high efficiency and less than 0.95 indicates low efficiency. The efficiency values are divided into four types (Fig 5).

"High PTE, High SE" refers to both the PTE and SE are higher than 0.95, and there are four provinces namely Shanghai, Jiangsu, Zhejiang and Liaoning in this type. This indicating that the internal management level and investment scale of UEBEI funds in these four provinces are relatively high, and they are in a leading position on TE. "High PTE, Low SE" refers to the PTE is higher than 0.95, the SE is lower than 0.95. Most of the provinces in China are in this type, although the urbanization level and the fiscal expenditure will be in a certain extent, promote the UEBEI’s efficiency increase, while the scale efficiency are still the key factors to constraint these provinces. "Low PTE, High SE" refers to the type with SE higher than 0.95 and PTE lower than 0.95. Guangdong and Sichuan are two provinces in this type, indicating that these two provinces need to further promote the PTE improvement, including improving the internal management level of the UEBEI. The income expansion of funds should be controlled and the level of fund operation should be enhanced. "Low PTE, Low SE" refers to the provinces with both PTE and SE lower than 0.95. Hubei is the only province in this type, indicating that both PTE and SE need to be improved. However, the reason may be that the COVID-19 outbreak has a relatively large impact on Wuhan’s economy and society, resulting in a temporary decrease.

## 6. Conclusion and enlightenment

This study uses a three-stage DEA model to analyze the efficiency of UEBEI fund expenditure in China, With fund income and the number of insured person as input indicators, fund expenditure and accumulated balance as output indicators, fiscal autonomy, the level of urbanization, the old-age dependency ratio and the level of marketization as environmental variables, and the comprehensive technical efficiency (TE), pure technical efficiency (PTE) and scale efficiency (SE) of 31 Provinces in China from 2016 to 2020 are calculated.

### 6.1 Conclusion

(1) From the overall data of the results in stage 3, the overall expenditure efficiency of the UEBEI in China is not high, and all provinces have not reached the efficiency frontier level, so there is still some space for efficiency improvement. From a regional perspective, the regional fund efficiency difference is significant and shows a pattern of East China (0.799) > Central China (0.725) > West China(0.548), that is according with the present situation of China’s economic and social development level. There is a big gap in resource endowment and development level between the East China and West China, and the eastern region is superior to the central and western regions. The pure technical efficiency in West China is relatively high, and the low comprehensive technical efficiency is mainly restricted by the low scale efficiency. Especially compared with the first stage, the decline trends of stage 3 in West China is unusually obvious, and there is still a large upside potential.

(2) It can be seen from the regression results of panel SFA in stage 2 that environmental factors and random disturbances have a significant impact on the operation efficiency of UEBEI fund, which may also include a series of negative collateral effects such as economic level fluctuations caused by COVID-19. The impact of environmental factors also seems to be more dominant. Therefore, the influence of environmental variables and random errors should be considered when using DEA model to calculate the actual efficiency. The regression results show that there is a positive correlation between the level of urbanization, the level of marketization and the expenditure efficiency of the UEBEI, while fiscal autonomy and the old-age dependency ratio are on the contrary which may be unfavorable to the improvement of the UEBEI’s expenditure efficiency to some extent. Compared to the efficiency value in stage 1, after eliminating the influence of environmental factors, the means of TE, PTE and SE all have a certain degree of volatility in each region. Especially the scale efficiency experienced a sharp decline in most provinces. The mean of TE is influenced by the common interacting of PTE and SE.

(3) The results indicate that considering the regional conditions differentiation, provinces and cities with high PTE and low SE, such as Beijing, Tianjin, Hebei and Fujian, should maintain the current level of allocation management and strive to adjust endowment insurance income scale to achieve the optimal scale. Provinces and cities with low PTE and high SE, such as Guangdong and Sichuan, should maintain the current financial input in pension security and improve efficiency by improving expenditure management and corresponding management ideas. The PTE and SE of Hubei province are both low, so it is necessary to increase investment in social security while improving fund allocation and management level after excluding the impact of COVID-19, improve the investment efficiency of UEBEI fund, and broaden the investment channels of the fund.

### 6.2 Policy implications

This study has certain management enlightenment and guiding significance to improve the expenditure efficiency of the UEBEI in China. In addition to implementing the differentiation strategy and improving the management level and fiscal scale according to local conditions, The government should pay more attention to the impact of environmental factors such as the level of urbanization, the level of marketization, fiscal autonomy and the old-age dependency ratio on the expenditure efficiency of the UEBEI. In order to further optimize the allocation of operational resources and improve efficiency of fund expenditure, we propose the following countermeasures and suggestions:

(1) Firstly, vigorously promote the construction of new urbanization, and promote equal access to public services across the country; Improve the infrastructure, in order to maximize the promoting role of urbanization on UEBEI. Urbanization is conducive to the long-term fiscal sustainability of the UEBEI, and the government should adhere to the "people-oriented" values to promote the construction of new urbanization. First of all, it should promote the orderly realization of citizenization of permanent residents who are capable of stable employment and living in cities and towns, including deepening the reform of household registration system, improving the labor skills and quality of migrant agricultural population, strengthening the basic public education guarantee for children of migrant agricultural population, and improving supporting policies etc. And next, the spatial layout and form of urbanization should be optimized to promote the coordinated development of large, medium and small cities and small towns, including improving the coordinated development mechanism of urban agglomerations and metropolitan areas, improving the function and quality of large and medium cities, enhancing the development vitality of small cities, and promoting urbanization construction with county seat as an important carrier. Third, accelerate the transformation of urban development mode, promote the construction of new urbanization. It includes increasing the supply of universal and convenient public services for the elderly, promoting the urban "aging-appropriate transformation" in an orderly manner, building a public health emergency prevention and treatment system, and advocating intelligent old-age care.

(2) Secondly, optimize the regional industrial structure, and reasonably promote the development of the tertiary industry; Take measures to promote and stabilize employment. In particular, government should focus on optimizing and developing the old-age service industry, promote the coordinated development of the old-age service industry and the public service for the old-age, and develop the “silver economy”. Meanwhile, it should unswervingly push forward the supply-side structural reform in the elderly care sector, combine diversified and personalized demand for healthy elderly care, promote the construction of the entire industry chain for elderly care, and promote technologicalized, intelligent and standardized elderly care services. In addition, it is also necessary to improve the employment rate in the fields related to the elderly, actively guide employees in new business forms to participate in the insurance, and increase the sustainability of pension. Specific measures may include continuing to energize market entities and ensure job creation, further unleashing the driving force of innovation and entrepreneurship and amplifying the multiplier effect of employment, ensuring the employment of key groups, such as social workers and old-age care workers, taking measures to encourage retirees reemployed, and ensuring that the bottom line of people’s lives is met.

(3) Thirdly, the government should promote the development of regional economy, reasonably control the ratio of fiscal revenue and expenditure of local governments, and accelerate national pooling of basic endowment insurance to improve the allocation and management of UEBEI funds. First of all, in light of China’s national conditions, it need to grasp the new development pattern of domestic circulation as the main body while domestic and international circulation mutually reinforcing, and achieve high-quality coordinated development under diversified strategic path among regions at a higher level. Specifically, it includes breaking regional division and eliminating all kinds of factors that hinder the flow of factors between regions, unblocking the economic circulation between the East and the West, and adopting the strategy of "Population flow East and Industry advance West", smoothing the economic circulation between the South and the North, promoting economic and technological cooperation between the two sides in various forms, and further shifting the focus of matching support to rural revitalization in underdeveloped areas etc. In the process of forming joint forces to accelerate regional integration, the government should fully implement the national pooling of endowment insurance funds. On January 1, 2022, China formally starts to put the enterprise employees’ basic endowment insurance into national overall planning, and the overall planning system has built and began to run. By the end of June 2022, the national centralized management of endowment insurance data and unified business risk control had been initially realized. Next it should continue to focus on promoting the national unified basic endowment insurance information system and multi-level social security information platform, accelerate the construction of standardization and normalization of social security, and improve the system application ability. By combining the traditional services and intelligent innovation, it will be more thoughtful and convenient for the old-age services.

(4) Fourthly, vigorously implement the strategy of active aging and gradually delaying retirement, and make concerted efforts by coordinating multiple subjects’ entities to achieve common prosperity in old-age security. Especially, Delayed retirement increases the time and amount of payment of endowment insurance premiums for employees and their companies, and shortens the length of time that the employees receive pensions, which is conducive to alleviating the imbalance between income and expenditure. However, delayed retirement is necessary to adopt a gradual delay scheme. First, the policy maker should respect the willingness of workers and delay the legal retirement age differently for different industries and types of job. For example, heavy manual workers such as coal workers are not suitable to delay retirement, while groups such as doctors and teachers may prefer to delay the legal retirement age. The second is to consider the implementation of flexible retirement system, which can not only give workers greater autonomy and humanistic care, but also avoid the waste of human resources. For example, setting a retirement age range. When the employee reaches the minimum retirement age, he or she can choose the appropriate time point after this age according to his or her own situation. When the maximum retirement age is reached, he or she must go through the retirement formalities. The third is to get a pension "reduced earlier and increased later", and implement incentives according to the actuarial balance of financial principles. That is, it is not necessary to force a certain age to retire, but those who retire early may receive a lower proportion of pension, and those who retire late can increase their pension by a certain proportion. Thus, incentivizing people to stay in work and voluntarily delay the retirement age.

## Supporting information

S1 Dataset (XLSX)
